# COVID-19 Vaccination Strategies in the Endemic Period: Lessons from Influenza

**DOI:** 10.3390/vaccines12050514

**Published:** 2024-05-09

**Authors:** Eliel Nham, Ji Yun Noh, Ok Park, Won Suk Choi, Joon Young Song, Hee Jin Cheong, Woo Joo Kim

**Affiliations:** 1 Division of Infectious Diseases, Department of Medicine, College of Medicine, Korea University, Seoul 02841, Republic of Korea; e.nham@kumc.or.kr (E.N.); jynoh@korea.ac.kr (J.Y.N.); okpark8932@korea.ac.kr (O.P.); cmcws@korea.ac.kr (W.S.C.); infection@korea.ac.kr (J.Y.S.); heejinmd@korea.ac.kr (H.J.C.); 2 Vaccine Innovation Center, Korea University, Seoul 02841, Republic of Korea

**Keywords:** COVID-19, influenza, vaccination, strategy

## Abstract

Coronavirus disease 2019 (COVID-19) is a highly contagious zoonotic respiratory disease with many similarities to influenza. Effective vaccines are available for both; however, rapid viral evolution and waning immunity make them virtually impossible to eradicate with vaccines. Thus, the practical goal of vaccination is to reduce the incidence of serious illnesses and death. Three years after the introduction of COVID-19 vaccines, the optimal vaccination strategy in the endemic period remains elusive, and health authorities worldwide have begun to adopt various approaches. Herein, we propose a COVID-19 vaccination strategy based on the data available until early 2024 and discuss aspects that require further clarification for better decision making. Drawing from comparisons between COVID-19 and influenza vaccination strategies, our proposed COVID-19 vaccination strategy prioritizes high-risk groups, emphasizes seasonal administration aligned with influenza vaccination campaigns, and advocates the co-administration with influenza vaccines to increase coverage.

## 1. Introduction

Coronavirus disease 2019 (COVID-19) is the first “Disease X” that was designated by the World Health Organization (WHO) as a blueprint priority diseases in 2018 [1]. It originated from wild animals and jumped to humans, is a primarily respiratory disease with remarkable transmissibility, and has considerable morbidity and mortality, especially in immunocompromised and/or unvaccinated individuals. After many efforts, including social distancing, mask wearing, the development of highly effective vaccines at unprecedented speed, and mass vaccination campaigns, the WHO declared that COVID-19 was no longer a public health emergency in May 2023 [2]. However, it is unlikely to disappear, like its close relative, the severe acute respiratory syndrome (SARS), which emerged in 2003.

All the characteristics listed above are similar to those of pandemic influenza. Novel pandemic influenza viruses originate from wild birds, affect hundreds of thousands of persons without prior immunity, and persist for an extended time, eventually becoming an “endemic”, seasonal disease [3]. After an unpredictable period ranging from 10 to 40 years, a new pandemic influenza virus emerges and follows a similar course.

The evolution of influenza vaccines has followed that of pandemic influenza viruses. After the 1918 pandemic, caused by an influenza A/H1N1 strain, a monovalent vaccine targeting H1N1 was approved in 1945 [3]. In 1957, the emergence of A/H2N2, a product of the genetic reassortment of avian and human influenza viruses, led to the development of the H1N1/H2N2 “bivalent” vaccine. Since the A/H3N2 influenza pandemic in 1967, the A/H2N2 component in the bivalent vaccine has been replaced by A/H3N2 [4]. Similarly, trivalent and quadrivalent vaccines have been developed after the widespread circulation of the influenza B/Victoria and B/Yamagata lineages in the late 1980s [5].

Breakthrough SARS coronavirus 2 (SARS-CoV-2) infections that occurred soon after COVID-19 vaccination led us to conclude that it was too optimistic to end the pandemic by achieving herd immunity. Despite several rounds of mass vaccinations over the last 4 years, sometimes with updated vaccines, the best strategy against this ever-changing virus remains unclear. In 2021, Dr. Monto suggested that experience with influenza vaccines may provide a more realistic perspective of this issue [6]. Therefore, based on added data since then, we discuss ways to achieve an optimal vaccination strategy for endemic COVID-19 and identify areas for further study.

## 2. Influenza and COVID-19: Similarities and Differences

To gain insights into vaccination strategies against pandemic and seasonal influenza, we summarized the similarities and differences between influenza and COVID-19.

### 2.1. Virus

#### 2.1.1. Subtypes and Variants

**Figure 1:**
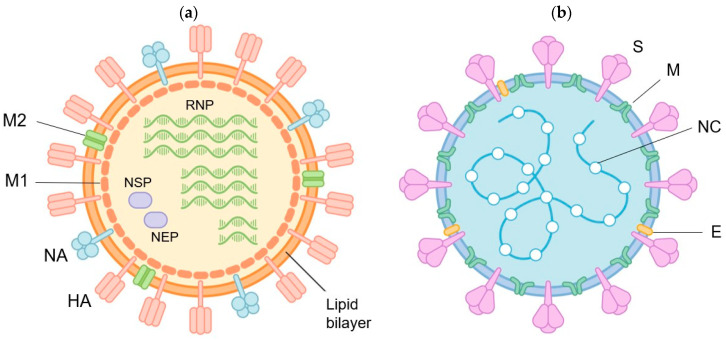
Structure of (**a**) the influenza virus and (**b**) SARS-CoV-2. Abbreviations: RNP, ribonucleoprotein; M2, matrix protein 2; M1, matrix protein 1; NSP, nonstructural protein; NEP, nuclear export protein; NA, neuraminidase; HA, hemagglutinin; S, spike protein; M, membrane; NC, nucleocapsid; E, envelope.

The influenza virus and SARS-CoV-2 are both enveloped RNA viruses that express several types of surface proteins crucial for infectivity (Figure 1). These include hemagglutinin (HA) and neuraminidase (NA) for the influenza virus and spike proteins for SARS-CoV-2. HA plays a significant role in viral attachment for host-cell entry, whereas NA is involved in the release of progeny virions after replication inside the host cells. The spike protein is the SARS-CoV-2-equivalent of HA.

Surface antigens are highly variable. Constant antigenic changes due to point mutations, mainly in HA and NA, lead to annual epidemics [3]. This is called “antigenic drift”. At longer but unpredictable intervals, influenza A viruses undergo more dramatic antigenic changes (“shifts”) through genetic reassortment with avian and human influenza viruses. These antigenic shifts generate antigens to which most of the population has no immunity and leads to pandemics. However, influenza B does not display similar antigenic variations in HA and NA as in influenza A [3,7]. In SARS-CoV-2, the accumulation of mutations in the spike protein has led to the emergence of new variants, causing subsequent epidemics.

The mutation rates of surface proteins are several to tens of times higher in the influenza virus than in SARS-CoV-2 [8,9,10]. However, this fact seems less relevant in the real world, where SARS-CoV-2 variants of concern with high infectivity and enhanced transmission emerge every few months. Kawasaki et al. explained this by highlighting the longer infection period and higher replication number of SARS-CoV-2 [10]. To date, there have been no reports of genetic recombination in SARS-CoV-2 similar to the antigenic shift in pandemic influenza; however, it is reasonable to assume its possibility [11].

#### 2.1.2. Disease Burden

Seasonal influenza is associated with 290,000–650,000 respiratory deaths annually [12]. COVID-19 seems to cause more severe disease than influenza in some populations, even after rounds of mass vaccination. In the 2022/2023 season, COVID-19 was associated with a higher risk of 30-day mortality in hospitalized patients (hazard ratio 1.61; 95% confidence intervals [CI], 1.29–2.02) [13]. A modeling study that estimated the mortalities associated with the Omicron variant and influenza infection also suggested a higher number of Omicron-associated deaths [14]. This trend has persisted through to the 2023/2024 season [15,16,17,18]. As of week one of 2024, 1.3% and 3.8% of mortalities have been attributed to influenza and COVID-19, respectively [15,17].

Both influenza and COVID-19 can result in various complications, such as pneumonia, secondary bacterial or fungal infections, respiratory failure, the acute exacerbation of underlying chronic medical conditions, myopericarditis, and inflammatory neurological diseases [19]. Because ACE-2 receptors are distributed throughout the body, SARS-CoV-2 infection causes a more severe systemic inflammatory response and more frequent multi-organ failure than influenza, which binds to sialic acid found only in respiratory epithelial cells. Lingering nonspecific yet disabling symptoms are far more commonly reported in patients with COVID-19 (“long COVID”) than in patients with influenza [20].

For both influenza and COVID-19, older adults, individuals with certain medical conditions, and pregnant women are disproportionately affected by severe infections.

- Older adults: Although the influenza incidence is higher in children than in older adults, morbidity and mortality are much higher in older adults [12,21,22]. This applies to both seasonal and pandemic influenza. Since the onset of the COVID-19 pandemic, older adults have accounted for a larger proportion of severe COVID-19 cases [23,24,25,26]. Between January and June 2023, adults aged ≥65 years accounted for 63% of COVID-19-associated hospitalizations and 88% of in-hospital mortalities in the United States (US) [26]. Much higher mortality rates are constantly reported in adults aged ≥75 years than in those aged 65–74 years. In December 2023, the monthly COVID-19 mortality per 100,000 was 22.6 and 3.6 in those aged ≥75 and 65–74 years, respectively [27].
- Persons with comorbidities: In people of all ages, the presence of chronic lung, cardiovascular, metabolic (including diabetes mellitus), neurological, or liver diseases is associated with an increased risk of intensive care unit (ICU) admission and mortality [23,28,29,30]. Obesity is another important risk factor [28,29,31]. Patients with immunocompromising conditions, such as various cancers, solid organ or hematopoietic stem cell transplants, or advanced human immunodeficiency virus infections, are among the most vulnerable [32,33,34].
- Pregnant women: During seasonal influenza epidemics and pandemics, pregnant women are more susceptible to severe influenza than non-pregnant women [35,36]. Influenza is also associated with adverse pregnancy outcomes [37,38]. Before the introduction of COVID-19 vaccines, pregnant women with COVID-19 were significantly more likely to be admitted to the ICU, require invasive ventilation or extracorporeal membrane oxygenation, or die compared with their non-pregnant counterparts [39]. Although transplacental transmission of SARS-CoV-2 is rare, COVID-19 is associated with pre-eclampsia, preterm birth, and stillbirth, especially in patients with severe disease [40]. This can be partially explained by the reduced accessibility to medical facilities during the pandemic [41]. However, to the best of our knowledge, no studies have compared the disease burden in pregnant and non-pregnant women since the introduction of the COVID-19 vaccine.

#### 2.1.3. Seasonality

Another difference between influenza and COVID-19 is seasonality. Influenza causes epidemics every winter in temperate regions and affects individuals in tropical regions year-round [3]. The disappearance of seasonal influenza was observed globally during the first 2.5 years of the COVID-19 pandemic; however, it largely reverted to a winter disease in the 2022/2023 season [42,43,44]. In contrast, COVID-19 outbreaks have not been limited to winter, which may be due to the higher transmissibility and rapid emergence of immune-evasive variants [45,46]. To date, it is uncertain whether SARS-CoV-2 will eventually circulate primarily between winter and early spring, similar to human coronaviruses [3,47]. Currently, COVID-19-associated hospitalizations and deaths are concentrated within this period when the burden of other respiratory pathogens is typically increased [48].

### 2.2. Vaccines

#### 2.2.1. Vaccine Effectiveness and Determining Factors

For influenza, vaccine effectiveness (VE) ranges from no effectiveness to as high as 60–70%, depending on the age, immune status, and health of the recipient; virus type and subtype; as well as the antigenic match between circulating virus strains and vaccine strains [3]. VE against influenza A/H3N2 tends to be lower than that against A/H1N1 and B. According to a meta-analysis that included studies conducted between January 2004 and March 2015, the pooled VE against laboratory-confirmed, medically attended influenza was 61% for A/H1N1pdm09, 67% for A/H1N1 (before 2009), 33% for A/H3N2, and 54% for B [49].

When COVID-19 vaccines were first introduced, their VE was higher than that of influenza vaccines: it was approximately 90% against symptomatic infections if a two-dose primary series with an mRNA vaccine or a protein-based vaccine was completed [50]. High VE against laboratory-confirmed, medically attended COVID-19 was maintained during the period when the Delta variant predominated [51]. However, studies have reported lower VE against later variants, i.e., Omicron (B.1.1.529) and its descendants, although protection against severe infections was less affected [52,53,54]. The decline in VE observed over time can be explained by waning immunity, limited cross-protection against immune-evasive variants, and immune imprinting.

- Waning immunity: For both influenza and COVID-19, immunity against symptomatic infections obtained through vaccination or natural infection begins to decline after several months [55,56,57]. This is mainly driven by a decrease in neutralizing antibody titers [58,59,60,61,62,63,64]. Protection against severe infections that lead to critical illness or death depends more on T cell responses, which last longer than neutralizing antibodies [3,65]. However, this wanes eventually, especially in individuals who are at higher risk of severe infection [57,66,67].
- Limited cross-protection: Persistent emergence of immune-evasive variants is another important reason for breakthrough infections. Since T cells mainly recognize internal antigens conserved across different variants, previous infection and/or vaccination confers some degree of cross-protection against severe infections caused by new variants [67,68,69].
- Immune imprinting: To address these challenges, both influenza and COVID-19 vaccines are regularly updated to contain antigens specific for the latest variants. However, even these efforts are complicated by the tendency of the immune system to boost immunity against previously recognized antigens rather than modified ones [70]. This presents as a poorer VE in people who receive annual influenza vaccinations than in those who do not [71]. Studies have reported, at best, modest boosting of neutralizing antibody titers against the Omicron variant by Omicron bivalent COVID-19 vaccines [72,73]. As the SARS-CoV-2 variants that emerged before Omicron are no longer circulating, creation of multivalent vaccines for COVID-19, such as those for influenza, is not required. Therefore, a monovalent formulation was used again for the 2023 updated vaccine.

#### 2.2.2. VE in Different Populations

While individuals at a higher risk of severe infections are the primary targets of vaccination, VE in these individuals tends to be lower. In this section, we summarize the data on VE against hospitalizations rather than symptomatic infections to focus on the impact of vaccines on more severe infections.

- Older adults: For influenza, the VE against hospitalization due to influenza and pneumonia is 25–53% [74]. Some studies have reported a similar VE between younger and older adults, whereas others have reported a lower VE in older adults [75,76]. Lower VE in older adults was more pronounced in A/H3N2-dominant seasons [49,77,78,79]. In the early phase of COVID-19 vaccinations, VE appeared to be similar between younger and older adults early after vaccination, but it declined faster thereafter in older adults [51,80,81]. However, in the era of Omicron predominance, VE in older adults was not lower than that in younger adults [66,82]. Following Omicron bivalent vaccination, the VE against COVID-19-related hospitalization among individuals aged 18–64 years was 61% within 60 days and 64% among those aged ≥65 years [66]. These figures decreased to 16% and 27%, respectively, after 120 days. The monovalent XBB.1.5 VE against hospitalization was 43% and 50% for those aged 18–64 and ≥65 years, respectively, within the first 4 months [82].
- Persons with immunocompromising conditions: According to a US Center for Disease Control and Prevention report, VE against influenza-related hospitalization among immunocompromised adults was 5% compared with the 42% in non-immunocompromised counterparts [83]. Another study reported a VE of 20% and 42% against hospitalization in patients with cancer and the general population, respectively [84]. For COVID-19, VE against hospitalization in patients with and without immunocompromising conditions was 63% and 91%, respectively, with a median of 42–44 days after a two-dose vaccination [85]. In the Omicron predominance period, the VE of a three-dose monovalent vaccination against COVID-19 hospitalizations was 69% within 90 days and 44% after 90 days in immunocompromised individuals [86]. These numbers were even lower during the BA.4/5 sublineage predominance period. No VE studies in this population were available after the introduction of Omicron bivalent or XBB.1.5 monovalent vaccines.
- Pregnant women: VE against severe influenza in pregnant women has rarely been studied. According to a study conducted in the 2018/2019 season, the influenza VE against influenza-like illnesses and hospitalization was 61% and 86.6%, respectively [87]. A meta-analysis that included studies published up to November 2022 found that COVID-19 vaccination significantly reduced the risk of COVID-19-related hospitalizations, ICU admissions, and stillbirths by 53%, 82%, and 45%, respectively [88]. Another study conducted during the Omicron predominance period (not included in the abovementioned meta-analysis) reported 48% and 76% VE against severe COVID-19 complications after two and three doses, respectively [89].

#### 2.2.3. Vaccine Safety

Both influenza and COVID-19 vaccines were developed to combat pandemics. Shortly after their introduction, the possible links between certain serious adverse events and the vaccines heightened public fear. The fact that influenza vaccination has been gradually accepted by the public, with continued safety evaluations, is a valuable precedent for COVID-19 vaccination.

An increased incidence of Guillain–Barré syndrome (GBS) in patients who received influenza vaccination was first noted during the 1976 pandemic. Since then, many studies have suggested a miniscule increase in GBS incidence after influenza vaccination, with approximately one additional case per million vaccinations [90]. During the most recent influenza pandemic, a similarly elevated risk of GBS was reported [90]. However, a multinational study did not observe any such associations after adjusting for confounders, including upper respiratory tract and gastrointestinal infections [91]. Although this association may be true, influenza vaccinations are still quite protective against GBS during an influenza season. According to studies which analyzed data between 1990 and 2011, the relative risk of GBS after influenza infection and seasonal influenza vaccination was 15.8–18.6 and 0.2–1.7, respectively [92]. Another condition that the AS03-adjuvanted H1N1pdm09 influenza vaccine (Pandemrix^®^) was associated with is narcolepsy [93,94]. However, later studies did not find a clear relationship between them [95,96].

The COVID-19 mRNA vaccine is associated with an increased risk of myocarditis or pericarditis [97,98]. This association is more pronounced in adolescents and young adults than in older adults and in males than in females [99]. The vast majority of cases were mild and self-limited [97]. In adults aged ≥65 years, myocarditis occurred in less than one in a million mRNA vaccinations over 120 days [100]. While a small increase in ischemic stroke incidence in Omicron bivalent vaccine recipients was reported, later studies found no such association [101,102,103]. To date, no other safety issues specific to older adults or immunocompromised patients have been reported.

#### 2.2.4. Vaccination Goals

The primary goal of influenza vaccination is to reduce morbidity and mortality by directly immunizing high-risk individuals, as well as to control outbreaks in the community. However, this alone is insufficient to control the disease burden. To control epicenters, the immunization of schoolchildren has become an important part of the influenza vaccination policy. Due to the low production costs, favorable safety profile, and socioeconomic burden of influenza, health authorities in some developed countries generally recommend that all persons aged ≥6 months be vaccinated annually.

For COVID-19, which is more transmissible than influenza, the goal of eliminating viral circulation through mass vaccination is unrealistic. Currently, most people gain immunity against COVID-19 through vaccination or natural infections. As of 10 February 2024, 65% of the world’s population have received primary-series vaccinations [104]. Meanwhile, all regions included in the WHO had already reached 90% seroprevalence as at April 2022, with many regions approaching 100% within the same year [105]. Most cases, including those that were once considered high-risk, typically exhibit only mild symptoms. Moreover, we have several effective antiviral drugs in our arsenal. Although mRNA vaccines are the most widely used COVID-19 vaccines, production costs are high. Therefore, reducing the incidence of severe infections and deaths among those at highest risk is the most reasonable goal.

## 3. Vaccination Strategy for COVID-19

### 3.1. Evolution of Vaccination Strategies

When COVID-19 vaccines were first introduced in late 2020, most nations had the same goal of immunizing everyone to end the pandemic by achieving herd immunity. Governments worldwide scrambled to vaccinate as many individuals as possible, ranging from those at the highest risk of severe infection to those at a lower risk [106]. Although most people have developed some degree of immunity, the waning of immunity and the recurrent emergence of immune-evasive SARS-CoV-2 variants have made it impossible to achieve herd immunity. Therefore, the primary goal has been modified to maintain immunity in high-risk groups. At this point, government vaccination strategies have begun to diverge according to their respective definitions of high-risk groups. This is affected by various factors, such as the population structure, social determinants of health, vaccine acceptance, vaccine availability, and the health system capacity. 

The latest vaccination strategies provided by different health authorities are summarized in Table 1. Notable points include the following recommendations: (1) single-dose vaccination in unvaccinated persons; (2) primary-series vaccination with an updated vaccine; (3) boosters primarily for specified risk groups (details differ by country); and (4) additional boosters in the spring.

### 3.2. Co-Administration of COVID-19 and Influenza Vaccines

COVID-19 booster doses are recommended once or twice a year for certain risk groups, preferentially in the autumn. As this strategy is similar to that for influenza vaccination, administering the two vaccines concomitantly would be helpful. This one-stop multiple vaccination strategy has already been implemented for other vaccines except in a few cases, where the simultaneous administration of two vaccines results in immune interference, decreasing the immune reaction to one or more vaccine components.

Studies on the co-administration of COVID-19 and influenza vaccines have shown conflicting results [107,108,109,110,111,112,113,114]. Some studies have reported significantly lower immunogenicity against SARS-CoV-2 or the influenza virus in the concomitant-vaccination group than that in the single-vaccination group (COVID-19 or influenza). However, not only do these “lower” immune reactions not necessarily translate to lower field effectiveness, but they can also be sufficiently high. One available effectiveness study found no significant differences in VE against COVID-19- or influenza-related healthcare encounters between the concomitant- and single-vaccination groups [115]. Although co-administration is indeed associated with a slightly reduced VE, it may still be beneficial for increasing vaccination rates and population immunity [116].

**Table 1:** Vaccination strategies across countries in 2023–2024.

Health Authorities	Target Groups	How to Vaccinate
World Health Organization [117]	High-priority group Adults aged >75–80 years * Adults aged >50–60 years * with or without comorbidities Adults with comorbidities (no mention of age) Subpopulation with special considerations Immunocompromised individuals Pregnant women Healthcare workers	If never received a COVID-19 vaccine: Immunocompromised individuals: two to three doses Pregnant women: one dose Others: one dose If received at least one dose of COVID-19 vaccine: Healthy adults, children, and adolescents: revaccination not routinely recommended Adults aged >50–60 years, adults with comorbidities, healthcare workers: revaccination 12 months after the last dose Adults aged >75–80 years or >50–60 years having comorbidities and immunocompromised individuals: revaccination 6–12 months after the last dose Pregnant women: one dose during each pregnancy
United States [118]	All individuals aged ≥6 months	From 6 months–4 years: Unvaccinated children: two or three updated doses (as primary series) Vaccinated children who have received one or two original or bivalent doses: one or two updated doses from the same manufacturer Vaccinated children who have completed a primary series: one updated vaccine dose, ≥2 months after the last dose Aged ≥5 years: One updated vaccine dose irrespective of previous vaccination history For moderate-to-severely immunocompromised persons: Unvaccinated individuals: three updated vaccine doses (as primary series) Vaccinated individuals who have received one or two original or bivalent doses: one or two updated doses Vaccinated individuals who have received ≥3 monovalent or bivalent doses: one updated dose. May receive additional updated doses ≥2 months after the last dose
Europe [119]	Adults aged >60 years Immunocompromised individuals Individuals with underlying medical conditions Pregnant women Healthcare workers	Starting in the autumn One or two updated vaccine doses according to risk A second dose offered ≥4 months after the last dose (adults ≥ 80 years or immunocompromised persons)
United Kingdom [120]	LTCF residents Adults aged ≥65 years Clinical-risk groups Immunocompromised individuals, their household contacts, and caregivers Healthcare workers and LTCF staff	Starting in the autumn One or two updated vaccine doses, according to risk: Two doses should be offered to older adults (≥75 years), LTCF residents, and immunocompromised individuals. A second dose offered around 6 months after the last dose (minimum interval: 3 months)
Australia [121]	Recommend for: Adults aged ≥65 years At-risk adults aged 18–64 years Consider in: Adults aged 18–64 years having no risk factors and at-risk adolescents aged 5–17 years Otherwise not recommended	Should be offered around 6 months after the last dose Additional doses recommended for adults ≥75 years, considered for at-risk adults aged 65–74 years, and for adults aged 18–64 years who are severely immunocompromised
Korea [122]	Recommend for: Adults aged ≥65 years Individuals with underlying medical conditions aged 12–64 years LTCF residents and staff	Starting in the autumn One updated vaccine dose irrespective of previous vaccination history, ≥3 months after the last dose

* Age cut-ff may vary by country. Abbreviations: COVID-19, coronavirus disease-2019; LTCF, long-term care facility.

Regarding safety, while a few studies have reported increased solicited systemic reactions in those who received concomitant vaccination, most were of a mild-to-moderate degree [107,108,109,123,124]. No other safety concerns were identified.

### 3.3. Our Suggestions

**Figure 2:**
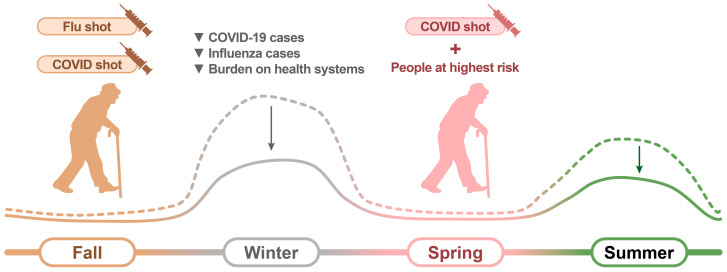
Scheme of the suggested COVID-19 vaccination strategy. The solid and dashed lines represent the expected volume of COVID-19 cases with and without vaccination, respectively.

Considering the aforementioned points, we propose the following COVID-19 vaccination strategy for the future (Figure 2):

- To whom: older adults (aged ≥65 years), individuals with immunocompromising conditions, and long-term care facility residents.
- When: every autumn, with an additional dose considered in the spring for individuals with the highest risk.
- What: monovalent, mRNA, or protein-subunit vaccine.
- How: co-administration with the influenza vaccine.

## 4. Discussion

Herein, we compare COVID-19 and influenza in terms of disease characteristics, disease burden, safety, and VE, using the data available to date. Based on this, we suggest a potential vaccination strategy for COVID-19 that can be applied in most high- and middle-income countries.

There are several lessons from the influenza vaccination programs. While some strategies, such as periodic vaccine updates and targeted vaccination campaigns at specific times of the year, are reflected in the current COVID-19 vaccinations, continuous efforts are required to address certain issues.

- Maintenance of an international virus surveillance system: This refers to being always prepared for the rise of highly evasive or virulent variants. The WHO has operated the Global Influenza Surveillance and Response System since 1952, which includes institutions in 129 WHO member states [125]. This enables the rapid collection and sharing of isolated viruses and information, including viral genetic sequences. Since the beginning of the COVID-19 pandemic, leveraging influenza surveillance systems for COVID-19 has played an invaluable role in the response to this novel pathogen [126]. Continued worldwide monitoring of SARS-CoV-2 and the prompt dissemination of genetic sequence data remain imperative. Information gathered through such systems also serves as a basis for the best vaccine strain selection.
- Establishment of correlates of protection (CoPs): CoPs are laboratory indicators that determine the presence of protective effects. They play an important role in the evaluation of vaccine products, the estimation of individual and population susceptibility to certain infectious diseases, and the validation of vaccines for which placebo-controlled trials are impractical or unethical [127]. For influenza, a hemagglutination inhibition (HI) titer of 1:40 is considered to provide 50% protection against influenza infection, though the HI assay is not without limitations [128,129]. The correlation between neutralizing antibody titers and the degree of protection against COVID-19 has been known since the early days of the pandemic. While some studies have suggested specific values, there remains no consensus on what neutralizing antibody levels guarantee a certain level of protection [130,131].
  It is necessary to present specific CoP (preferably quantitative and functional) indicators by correlating them with clinical data through standardized analyses from reliable institutions. COVID-19 has a higher disease burden than influenza, and the cost-effectiveness of vaccination in an epidemic situation has not yet been fully revealed; therefore, knowing the CoP of vulnerable population groups will be beneficial in establishing vaccination policies. Additionally, because updated COVID-19 vaccines are distributed without efficacy trials, the use of CoPs is desirable to verify in advance whether the vaccines are sufficiently effective.
- Need for mucosal vaccines: An influenza nasal spray vaccine (Flumist^®^) is available for immunocompetent people aged 2–49 years [132]. The spray contains live attenuated influenza viruses that inoculate the upper respiratory mucosa and induce mucosal immunity. The pain-free nature of nasal spraying is welcomed by children, for whom the effectiveness of the mucosal influenza vaccine seems to be the greatest [3]. The VE of the nasal spray vaccine is maximized during well-matched seasons, effectively reducing viral transmission in the community [3]. The complementary use of mucosal vaccines could also be useful for preventing COVID-19, as systemically administered inactivated vaccines are less effective in inducing mucosal immunity and consequently in inhibiting viral transmission. In addition to the two locally approved intranasal vaccines in China and India, other candidates are undergoing clinical trials [133,134].

Some points should be considered regarding our suggested vaccination strategy. First, annual boosters in fully vaccinated adults aged 65–79 without comorbidities may not be cost-effective in many countries, as the risk differential compared to younger adults <65 years is relatively modest [25,26]. However, we suggest including this age group, because it would make it practically easier to promote co-administration with the influenza vaccine, which is recommended for adults aged ≥65 years. Second, our strategy is based on data up to early 2024, when the descendants of the Omicron variant are still dominant and no other variants with greatly increased immune evasiveness or morbidity have been identified. The target population may be expanded if such variants emerge in the future, e.g., to include healthcare workers who can transmit the virus to high-risk populations. Third, due to the sheer volume of literature on mRNA vaccines, our recommendations were derived primarily from mRNA vaccine studies. Although they are useful for the rapid adaptation to emerging variants, such expensive vaccines may not be affordable or cost-effective in low-income countries. Therefore, protein-subunit vaccines are a reasonable alternative. Fourth, vaccine hesitancy is a crucial issue to be addressed. Although this subject was not discussed in this review, health authorities should continue to deliver appropriate information and clear messages toward target populations.

In conclusion, we compared COVID-19 and influenza based on the available literature to obtain insights from previous experiences with pandemic and seasonal influenza vaccination. Our COVID-19 vaccination strategy is applicable to a wide spectrum of countries. However, considering the dynamic nature of the situation, it is crucial to stay prepared for potential changes that may arise.
